# Detecting variable responses in time-series using repeated measures ANOVA: Application to physiologic challenges

**DOI:** 10.12688/f1000research.8252.2

**Published:** 2016-07-08

**Authors:** Paul M. Macey, Philip J. Schluter, Katherine E. Macey, Ronald M. Harper

**Affiliations:** 1 UCLA School of Nursing, LA, CA, USA; 2 Brain Research Institute, Department of Neurobiology, David Geffen School of Medicine, UCLA, LA, CA, USA; 3 School of Health Sciences, University of Canterbury, Christchurch, New Zealand; 4 Department of Neurobiology, David Geffen School of Medicine, UCLA, LA, CA, USA

**Keywords:** mixed effect models, regression analysis, statistical models, physiological responses, functional magnetic resonance imaging

## Abstract

We present an approach to analyzing physiologic timetrends recorded during a stimulus by comparing means at each time point using repeated measures analysis of variance (RMANOVA). The approach allows temporal patterns to be examined without an *a priori *model of expected timing or pattern of response. The approach was originally applied to signals recorded from functional magnetic resonance imaging (fMRI) volumes-of-interest (VOI) during a physiologic challenge, but we have used the same technique to analyze continuous recordings of other physiological signals such as heart rate, breathing rate, and pulse oximetry. For fMRI, the method serves as a complement to whole-brain voxel-based analyses, and is useful for detecting complex responses within pre-determined brain regions, or as a post-hoc analysis of regions of interest identified by whole-brain assessments. We illustrate an implementation of the technique in the statistical software packages R and SAS. VOI timetrends are extracted from conventionally preprocessed fMRI images. A timetrend of average signal intensity across the VOI during the scanning period is calculated for each subject. The values are scaled relative to baseline periods, and time points are binned. In SAS, the procedure PROC MIXED implements the RMANOVA in a single step. In R, we present one option for implementing RMANOVA with the mixed model function “lme”. Model diagnostics, and predicted means and differences are best performed with additional libraries and commands in R; we present one example. The ensuing results allow determination of significant overall effects, and time-point specific within- and between-group responses relative to baseline. We illustrate the technique using fMRI data from two groups of subjects who underwent a respiratory challenge. RMANOVA allows insight into the timing of responses and response differences between groups, and so is suited to physiologic testing paradigms eliciting complex response patterns.

## Introduction

We describe a procedure for analyzing physiologic signals recorded during a stimulus at each time point across multiple subjects without an *a priori* pattern or timing of expected response. The concept is to average the signal overall stimuli and over subjects, and look at differences between groups and conditions, especially the interaction with time since onset. The time of onset is treated as a baseline that is followed by one or more stimuli and recovery periods, a paradigm common to physiologic challenges. Because the challenges are clustered within subjects, the data are modeled with a mixed linear model. The result is a figure with the average time course per group/condition, with a statistical test comparing the signal at each time point. The model tested is then SIGNAL = GROUP + TIME + GROUP*TIME. Other covariates may be added. Importantly, the TIME variable is modeled as categorical, not continuous. Modeling time as categorical allows tests for each time point to be performed separately in post-hoc testing. The results of interest are the post-hoc tests of the interaction effect between group and time.

We initially designed this approach to analyze functional MRI (fMRI) time series recorded during gas and blood pressure challenges, such as hyperoxia, cold pressor, and Valsalva tests ^1– 3^. The protocol for such tests is the typical “boxcar” model, with a baseline period followed by one or more challenges, followed by recovery periods. We subsequently applied the method to heart rate and respiratory signals recorded during physiologic challenges ^4^. Functional MRI analyses usually require defining a model and identifying voxels whose time courses follow that model; other model-free event-related approaches test for responses that are coincident in time or duration. However, certain tasks elicit neural responses that vary in pattern and timing. For example, responses to physiological challenges typically occur across neural structures in a sequence rather than simultaneously, and the patterns are not “on/off” but differ according to structure ^5– 7^. While existing software like SPM does allow for model-free assessment of responses, such as identifying the hemodynamic response based on a finite impulse response function in SPM ^8^, those approaches can be complex to implement and interpret, especially for multiple groups and longer challenges. The present method arose out of our need to perform basic time-trend analyses of fMRI signals in specific regions.

We first developed the approach to better understand the nature of differences detected by traditional whole-brain fMRI analyses such as implemented in SPM. Typical whole-brain results consist of significance maps indicating areas of signal increase or decrease, or perhaps of group differences, but identifying the exact nature of such differences requires extracting and analyzing underlying timetrends. In other words, SPM gave us “blobs” where brain function had varied from baseline, but we wanted to know exactly how and when those fMRI responses changed. In physiologic regulation, timing is critical, and we aimed to identify specific time-points at which fMRI signals significantly increased or decreased following a challenge, and points at which group differences emerged.

The approach we propose is analyzing time series using repeated measures analysis of variance (RMANOVA), allowing for detection of within-group responses relative to a baseline or resting state, and of between-group differences in response. As with any analysis of variance (ANOVA), RMANOVA tests the equality of means that are assumed to be normally distributed. However, RMANOVA also accounts for the correlation between repeated measures within subjects, whereas ANOVA does not. In addition to assessment of significant reactions relative to a baseline period, or of significant group differences in response, the method allows for identifying specific time-points when these differences occur. Therefore, transient, late-developing, and complex sequences of increasing and decreasing signal changes can be distinguished with RMANOVA. The technique is suited to analysis of longer task paradigms evoking a complex pattern of responses. We present implementations of this form of RMANOVA in R ( https://www.r-project.org/) and SAS (SAS Institute Inc., Cary, NC).

## Description of procedure

Functional MRI data collected in one or more groups of subjects are preprocessed, typically with motion correction, and the images may be spatially normalized. An additional, optional step is detrending to remove global effects. In traditional cluster analyses, global effects may be accounted for within the analysis steps using, for example, SPM ^9^. For the example data, removal of global signal changes was performed on the preprocessed images prior to analysis with RMANOVA using a custom technique ^10^. Physiologic data that are direct measures such as heart rate do not need preprocessing.

For fMRI analyses, volumes-of-interest (VOI) are defined, usually by outlining a structure of interest on an image volume and saving that outline as a binary image, or mask. The VOI may be defined individually on a subject-by-subject basis, or globally for all subjects on a template (the latter case assumes subjects’ images are accurately spatially normalized to the template). The mask is used to extract the intensity of only those voxels within the VOI, at each time point in the fMRI series. For each time point, the average of those voxel values is recorded, resulting in a time-series corresponding to the blood oxygen level dependent (BOLD) signal time course of that VOI in the fMRI series. The procedure is repeated for all subjects, resulting in one time trend for each subject per VOI. The time trends may optionally be deconvolved with a hemodynamic response function (HRF), allowing the timing of significant responses to be inferred as neural, rather than BOLD. The resulting VOI time trends are subjected to a RMANOVA. Physiologic data measured at non-uniform intervals may need to be resampled so that each subject has a measure at equivalent time points.

The repeated measures ANOVA is using a mixed effect model, a generalization of the standard linear model which allows for correlation and non-constant variability within the data ^11^. Common RMANOVA implementations such as “aov” in R and “ranova” in MATLAB require the same number of measurements for each time bin, which is not always possible with data from physiologic challenges. In the mixed model implementation of RMANOVA, each subject is included as a random factor, together with a covariance structure that accounts for repeated measures. The methodology described here was originally based on the SAS function PROC MIXED, and extended to the R procedure “lme” in the “nlme” library. The SAS approach is flexible, and includes a wide range of diagnostics tests. (See white paper “Comparing the SAS® GLM and MIXED Procedures for Repeated Measurements Analysis,” Russ Wolfinger and Ming Chang, SUGI Proceedings, 1995; available at https://support.sas.com/rnd/app/stat/papers/abstracts/mixedglm.html.) The procedures calculate the variance-covariance matrix based on the dependencies defined by repeated measurements within subjects, and by group classification of subjects in the case of more than one group (REPEATED and GROUP options within PROC MIXED; by convention, SAS syntax is in upper case) ^11^. There is no R equivalent of PROC MIXED, so a series of functions are required. The “lme” function is more limited than PROC MIXED, but nevertheless is sufficient for implementing the core model in the present application.

A classification variable ( *t*) representing baseline and time-points in the task or challenge period is created, with values of *t* during the baseline or reference period constant, and values increasing for each time point in the series. For example, we have collected fMRI data during physiological challenges with one minute of baseline followed by 90 sec of challenge, at a repetition time of 6 seconds (25 volumes total); thus, *t* is 0 for the first 10 scans, and 1 for the 11 ^th^ volume, 2 for the 12 ^th^, and so on until 15 for the 25 ^th^. Note that for data collected at higher sampling rates or across longer time periods, the time bins may be increased, and the time-classification variable may include multiple data points, as opposed to the individual data points of the fMRI time-series; for example, we have classified instantaneous (beat-by-beat) heart rates recorded throughout a two minute period into twelve 10 sec epochs ^12^. If more than one group of subjects is being analyzed, a second classification variable is defined at each time point with a value representing the group to which that series belongs. The data format therefore consists of column variables of the response, time bin, subject, group, and optionally other covariates. Note this format differs from simpler implementations of RMANOVA which require one row with responses for all time bins.

Before assessing the time-trend results of the RMANOVA analysis (see sections below for full details), certain residual and influence diagnostic tests should be performed to ensure that the model does not seriously violate underlying assumptions. A relevant subset of such tests is described in sections below. Many practical applications of RMANOVA violate the model assumptions to some degree, but the approach is considered robust to moderate departures of these assumptions. In the present application to physiologic data, a key test is to ensure that the residual distributions are not seriously skewed. We suggest the flowing checks. First determine the studentized residuals, which are scaled so that more than 95% should fall within the critical limits of the appropriate Student’s t distribution, which for most purposes can be assumed to be within ± 1.96 (and centered on 0). The shape of the histogram for each group of the studentized residuals should be bell-shaped. There should also be no effect of time, so each group plot of the studentized residuals by time should not show a trend. We recommend using locally-weighted-scatter-plot (lowess) curves to identify such trends. In the case where the diagnostic tests fail, there may still be some value in continuing with the model, but we suggest presenting the results of the residual diagnostics along with the findings, to allow readers to assess for themselves the validity of the model. Further options are explained below, along with other diagnostic tests. Because these tests are closely tied with the software implementation, they are described in conjunction with the code.

There are three levels of results produced by RMANOVA: the significance of the overall model, the significance of the independent variables and their interactions at the group, time, and group-by-time levels, and (for post-hoc testing) the significance of between-group and within-group effects at each time point. The sequence is to test 1) global fit of the model; if significant then test 2) variable and interaction level fit; and if significant test 3) post hoc at individual time points. That is, by the Tukey-Fisher criterion for multiple comparisons, the model is first assessed for overall significance before investigating time-point specific effects; i.e., if the overall group-by-time effect is not significant, the between-group effects at each time point are not considered, regardless of their reported significance level. Similarly, if the overall time effect is not significant, then within-group effects at each time point are not considered.

Specific programming details are presented in the next sections to facilitate replication.

### Text file format for exporting to R and SAS

Data are arranged in columns with one row per observation. Each row has the time bin (“epoch”), response value (“y”), and subject. For more than one group, a group value is required, and additional covariates can also be included. Each subject is identified by a unique classification variable (SUBJECT), and similarly, the group to which that subject belongs is designated by a GROUP classification variable e.g., 1, 2, or CONTROL, OSA). For any subject, multiple values for each epoch may be included, and different numbers of observations for each epoch are allowed; the baseline will usually have the most observations. Values for fMRI are typically in percentage change relative to the baseline period.

The SAS code for importing these data in a text file “fn” with a header row into a library “voilib” is as follows:

LIBNAME voilib ‘[ **sas_library_folder**]’;

PROC IMPORT OUT=voilib.data

DATAFILE = '[ **fn**]'

DBMS=TAB REPLACE;

GETNAMES=YES;

DATAROW=2;

RUN;

here [ **sas_library_folder**] is the path to where the SAS library files will be stored. Note that SAS code is not case dependent. In the examples presented here, capitals are used to indicate SAS commands, and bolded names within square brackets are used to indicate user-specified variables. The remaining lowercase names are variables specific to these examples.

The R code is as follows:

setwd(“[ **R_library_folder**]”)

voidat <- read.table(“[ **fn**]”, sep="\t", header=TRUE)

### RMANOVA as mixed model

The SAS RMANOVA, along with formatting and output filtering, is implemented as follows:

PROC MIXED noitprint noclprint data= voilib.data empirical;

*class group epoch subject;*

model y = group epoch group*epoch / outp = prediction RESIDUAL;

*repeated / type=cs sub=subject(group) group=group;*

lsmeans group*epoch / DIFF;

ods listing exclude lsmeans;

ods output lsmeans= voilib.means;

ods listing exclude diffs;

ods output diffs=voilib.differences;

ods output CovParms= voilib.covparams;

ods output FitStatistics= voilib.fitstatistics;

ods output Tests3= voilib.type3tests;

RUN;

where the italicized statements define the model, and the remaining control the output (including residuals). Note that the RESIDUAL option, which directs studentized residuals to be calculated, is only available from SAS version 9.1. The CLASS statement determines classification variables. The EMPIRICAL option in the proc mixed command line uses the Huber-White sandwich estimator of variance, rather than the default, as it is more conservative but considered more robust, particularly if the assumption of normality is violated (as is often the case).

The R model first requires converting the classification variables to factor objects:

fmridat <- within(fmridat, {

epoch <- factor(epoch) # time was continuous but for RMANOVA must be categorical

subject <- factor(subject)

group <- factor(group)

})

The mixed model is then implemented in lme with the option to fit a random intercept per subject, which is equivalent to PROC MIXED with compound symmetric covariance matrix.

library(nlme)

out.lme <- lme(y~group*epoch, random = ~1 | subject, data= fmridat)

Although other covariance matrices other than compound symmetric might in theory be more suited to the present application, in our tests with SAS we found greatly increased computational time for minimal differences in results (for example with compound symmetric heterogeneous), so the proposed option in R should be suitable for the current application.

In SAS, the output from this step contains numerous descriptors of the model. The “Null Model Likelihood Ratio Test” gives the probability of the null model being true (column heading “Pr > ChiSq”). If the model passes the Tukey-Fisher criterion for multiple comparisons, i.e., the probability of the null model is less the 0.05, the model is investigated at the variable level using the “Type 3 Tests of Fixed Effects’ output. Probabilities of the null contribution to the model by individual independent variables are presented in the “Pr > F” column for the group, epoch and group*epoch. “Group” represents a group effect, which is often not significant for fMRI data presented in percent change relative to baseline. The variable epoch represents a time effect, corresponding to within-group significant responses. The group*epoch term represents a group-by-time effect, corresponding to between-group differences across time. Again considering the Tukey-Fisher criterion, if for any of these variables the likelihood of the null model is greater than 0.05, then that variable is determined to be not significant. If less than the threshold, the model is investigated further at each time point.

For R, further commands are required to generate equivalent statistics. The “summary(out.lme)” will provide global level fit statistics (at the start of the output listing). The ANOVA table created with “anova(out.lme)” will provide the separate group, group*epoch, and epoch statistics.

Further code is needed to select the relevant output for determining the time points of within and between group significant differences. The SAS code below is one of several possible ways to extract the relevant results.

The following command keeps all comparisons at the same epoch, hence the between-group table. The SORT step orders the table.

DATA voilib.betweengroup; SET voilib.differences;

IF epoch ne _epoch THEN delete;

DROP _epoch;

RUN;

PROC SORT DATA= voilib.betweengroup; BY epoch group; RUN;

The following command extracts the within group table. Between group comparisons are dropped, and only those relative to baseline (epoch 0) are kept. The SORT step orders the table.

DATA voilib.withingroup; SET voilib.differences;

IF group NE _group THEN delete;

DROP _group;

IF epoch NE 0 THEN delete;

ROC SORT DATA= voilib.withingroup; BY group _epoch; RUN;The above code will create Withingroup and Betweengroup tables in the SAS library voilib.

In R, the “Post-Hoc Interaction Analysis” (phia) simplifies the equivalent process. For between-group tests at each epoch the following code will export the results to a text file.

library(phia)

num_group <-length(unique(fmridat$group))

if (num_group>1) {

   # Between group is easy

   bg <- testInteractions(out.lme,pairwise="group",fixed="epoch")

   write.table(bg,file = "betweengroup.txt", sep = "\t")

}

The withingroup results require extracting only the differences of non-baseline epochs relative to the baseline (epoch=0), within groups. The following code is one approach.

library(phia)

wg <- testInteractions(out.lme,pairwise="epoch",fixed="group")

# However, only interested in responses wrt baseline, so extract epoch 0 vs others.

# E.g., we are not interested in comparison between epoch 5 and 10.

# Number of time bins

num_epoch <- length(unique(fmridat$epoch))

grouplist = unique(fmridat$group)

# Use the "data.table" alternative to R frame - apparently faster, less memory intensive, and simpler coding.

library(data.table)

dattable = data.table(fmridat)

# The number of subjects is calculated for each group.

# Initialize vector (required by R)

num_subjects <- vector(mode="numeric", length=num_group)

for (i in 1:num_group) {

groupdat <- dattable[group == grouplist[i]]

num_subjects[i] <- length(unique(groupdat$subject))

}

# Create logical vector to isolate the first entries (epoch 0 vs...) for each group

# The "wg" withingroup table has all combinations of epochs for each group

# Calculate the number of combinations

num_epochcomb <- choose(num_epoch,2)

# Initialize true/false vector; default is false

ind2baseline_wg <- vector(mode = "logical", length=num_group*num_epochcomb)

row_upto <- 1

for (i in 1:num_group) {

row_1 = row_upto;

row_2 = row_upto + num_epoch-2;

ind2baseline_wg[row_1:row_2] <- TRUE

row_upto <- row_upto + num_epochcomb

}

# Using logical vector, create table with only epoch 0 vs others (for each group)

wgb <- wg[ind2baseline_wg,]

write.table(wgb,file = "withgroup.txt", sep = "\t")

**Figure 1.**
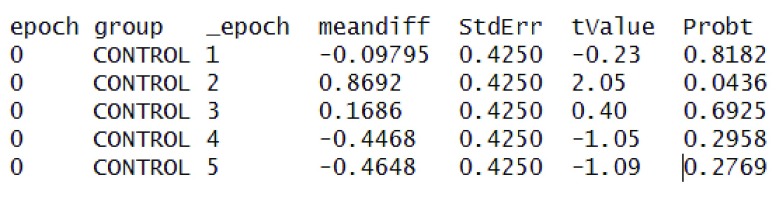
Example of within-group table of time-points of difference relative to baseline (epoch 0).

If “epoch” showed a significant effect in the Type 3 tests (above), the time points where this difference appeared may be determined by examining the withingroup table. As shown in Figure 1, each row in the table compares baseline (labeled “epoch”) vs subsequent epochs (labeled “vs epoch”), with a mean difference, standard error and P value; if the latter indicates significance (< 0.05), that time point is considered to have a significant response relative to baseline for that group.

**Figure 2.**
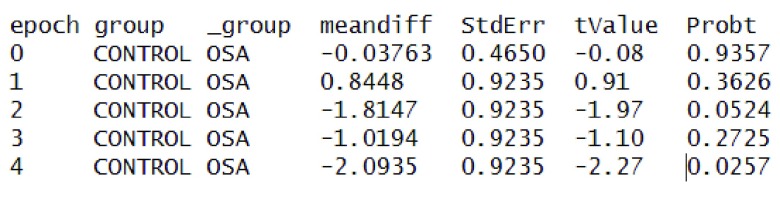
Example of between-group table of time-points of difference between groups.

Similarly, if “group*epoch” showed a significant effect in the Type 3 tests (above), the time-points where this difference appeared may be determined by examining the Between group table. As shown in Figure 2, each row corresponds to a time point (labeled “epoch”) and compares one group vs the other, with a mean difference, standard error and *P* value; if the latter indicates significance (< 0.05), that time point is considered to have a significant group difference.

### Assumptions, diagnostic checks, and limitations

The RMANOVA method has a number of underlying assumptions which should be considered. The assumptions of the RMANOVA method presented here which differ from ANOVA are that 1) the means are linear over time, 2) multivariate normality, 3) homogeneity of covariance matrices, and 4) independence. The method is reasonably robust to violations of the second and third assumptions. Violations of independence result in non-normal distributions of the residuals, which invalidate the F-ratio. While violations of independence in time series regression models arise due to correlations between errors at different time points, in RMANOVA the problem would arise if the model systematically over- or under-predicts for particular independent variable values. The most common violations of independence occur when either random selection or random assignment is not used or the compound symmetry covariance assumption is inappropriate. Violation of the homogeneity of covariance matrices generally results in the overall test having a higher Type I error rate than nominally set.

In the current application, a subset of key diagnostic and residual checks is suggested with the implementation of this technique.

- Plots of predicted and observed data, to visually determine any major bias.
- Residual checks of normality, including 95% of Studentized residuals falling within ±1.96, showing approximately normal group distributions, and scatter plots over time with lowess curves superimposed to ensure lack of group trends with time. Formal tests of normality may also be performed, although these will typically fail.

Assuming the “RESIDUAL” option in PROC MIXED has been used, the output will show these plots.

**Figure 3.**
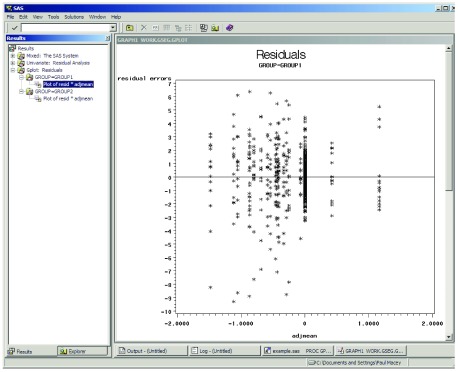
Plot of residuals for one group.

**Figure 4.**
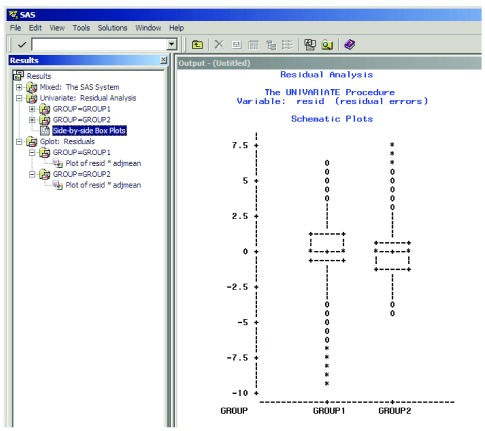
Box plot of residuals for both groups.

The quantitative tests of normality in the proc univariate results give likelihoods of the each variable being normally distributed; for multivariable normality, all variables should be normally distributed, although this assumption is usually violated to some degree. The output of the UNIVARIATE procedure includes histograms of residuals (for assessing whether shape of distribution is approximately normal) and side-by-side box plots of residual distribution by group ( Figure 3 and Figure 4). In R, the lme object has diagnostic plot methods including boxplots, by subject and by group in the following examples:

plot(out.lme, subject ~ resid(.)) # by subject

plot(out.lme, group ~ resid(.)) # by group

### Tests of influence

Further possible tests include influence diagnostics to determine whether particular subjects have undue effect on the model. If undue bias due to individual subjects is suspected, the approach we suggest is to use the influence measures to investigate whether one or more subjects are consistently and substantially discrepant from the others. In SAS these tests may be run by adding the INFLUENCE statement [e.g., after in PROC MIXED after “RESIDUAL” add “INFLUENCE(EFFECT=subject ITER=5)” and ODS SELECT INFLUENCE]; note that this requires SAS version 9.1 or greater. This test produces the restricted likelihood distance, predicted residual sum of squares (PRESS) statistic, and Cook’s distance (known as Cook’s D) for each subject. In R, one option is to use the “predictmeans” package to calculate Cook’s D and display the standardized residuals, and the PRESS statistic may be calculated directly from residuals predicted using the original data, as shown below.

library(predictmeans)

CookD ( out.lme, group = "subject" ) # where out.lme <- lme(…)

residplot(out.lme)

pr <- residuals(out.lme)/(1 - predict(out.lme,dat2))

press <- sum(pr^2)

At the time of writing, there is no equivalently simple R function to calculate studentized residuals for an lme mixed model.

If a discrepant subject is found, an attempt can be made to determine whether there is some reason for this. If a reason can be found, then they might legitimately be excluded. A “sensitivity” analysis may be run (a comparison analysis including and excluding that patient) to see if there is a material difference in the conclusions or results of interest. In SAS, the homogeneity of covariance matrices can also be assessed in a similar manner using the ESTIMATES option in the INFLUENCE statement. Although these influence diagnostics may be useful in some cases, they have less relevance in fMRI analyses due to the controlled nature of typical experiments and are presented here as optional.

### Options if diagnostic tests fail

If the residuals are non-normal but symmetrical around 0 then the model is probably robust. However, if the residuals are both non-normal and non-symmetrical (i.e., skewed) then estimate biases may appear. For a large number of patients or observations, results are typically robust to moderate amount of skew. For smaller numbers, even a smaller amount of skew will mean the model is unlikely to be robust. If the data are seriously skewed, the typical options include making transformations (log, power transformations, etc.), identifying and omitting unusual patients (see above), using another more appropriate analytical technique, introduce covariates into the model, or not analyze the data. For fMRI studies, omitting unusual patients and adding covariates are the most likely approaches. As mentioned earlier, if the findings from the model are considered of value, then we suggests researchers report them along with the diagnostics test results so that the reader can assess for themselves the validity of the results.

### Additional limitations

RMANOVA has additional limitations which may be less applicable to fMRI data, but are detailed below for completeness. The constraints of the assumptions result in weaknesses of the approach, including:

- Missing time bin data, potentially relevant to non-fMRI data such as beat-by-beat measures of heart rate, requires cases to be deleted from the analysis, causing both conceptual and analytical difficulties. Imputation methods can be employed to circumvent this issue. However, for any time bin subjects may have differing numbers of responses. For example, at “epoch 5” subject 1 may have a single response whereas subject 2 may have three measures; such a scenario could occur with breath-by-breath respiratory rate responses.
- Tests of within-subjects effects assume sphericity, but this was not directly evaluated. The data can be tested for sphericity, but this evaluation is complex within the proc mixed framework, and is not addressed here. In theory, Mauchly’s test of spherericity can be used, for example, when any within-subjects factor has three or more trials. (If the within-subject factor fails to meet the assumption of sphericity, then either the multivariate approach can be used or the univariate results can be adjusted using a correction factor, e.g., the Huynh-Feldt Epsilon correction method [the Greenhouse-Geisser method has been shown to be too conservative]).
- The choice of the within-subject correlation matrix form is dependent upon sphericity assumptions, and we suggest a compound symmetric matrix (“type = cs” option in proc mixed; random intercept per subject as suggested for R is equivalent) to allow for different variance patterns across groups. If different variance patterns across time are suspected, then heterogeneous compound symmetry could be used (“type = csh” option in SAS; not available with suggested R implementation). However, this process is extremely computationally intensive, and in tests we performed, made no notable difference to the fit of the model.

Finally, in SAS the library voilib created with the above code contains numerous tables of data including predicted values, observations, model fit statistics, and residuals. These tables can be examined further.

## Implementation and results

### Test data and subjects

We used fMRI data collected from two obstructive sleep apnea and two healthy control subjects as part of a pilot project; data are available online ^9^. The data were chosen to illustrate the RMANOVA methodology, and do not constitute a sample that is sufficient to test scientific hypotheses. The Institutional Review Board of UCLA approved this study (IRB# 10-001012), which was in compliance with the Declaration of Helsinki; participants provided informed, written consent after the nature of the procedures was explained. Following a baseline scanning period, subjects performed a series of four respiratory challenges (30 s maximal inspiratory apnea), which were expected to elicit abnormal physiological responses in the patient group, based on earlier demonstrations of impaired neural responses to physiologic challenges ^1, 13– 15^. A standard fMRI whole-brain protocol with repetition time of 2.5 s was implemented on a 3T Siemens Trio MRI scanner, and high-resolution T1-weighted anatomical scans were also collected (voxel size = 0.9 × 0.9 × 1 mm). The fMRI data were preprocessed using SPM routines, including realignment, spatial normalization, and smoothing. Smoothed images were intensity normalized to minimize global effects. VOI derived from the “AAL” toolbox in SPM were used to extract timetrends from the processed data ^16^, and eight were selected to illustrate a variety of patterns highlighted by the approach. For each VOI, the time-series were analyzed using RMANOVA across the groups of two subjects, with challenges combined. Accompanying SAS and Excel files for each VOI are included online with this publication (Data availability).

Processed data, as well as the SAS code for running each VOI analysis Organised as subfolders for each VOI, labeled by figure section. The VOI itself is also included as a nifti file, and in a pdf file overlaid onto a normalized background. Click here for additional data file. Copyright: © 2016 Macey PM et al. 2016 Data associated with the article are available under the terms of the Creative Commons Zero "No rights reserved" data waiver (CC0 1.0 Public domain dedication).

Preprocessing was performed using MATLAB 7 (The Mathworks, Inc., Natwick, MA, USA), SPM ( http://www.fil.ion.ucl.ac.uk/spm/) and a custom detrending routine ^10^. VOI were drawn using MRIcroN software ^17^, and RMANOVA was implemented in SAS 9.4 ^11^.

### Evaluation of fMRI responses, within and between groups

**Figure 5.**
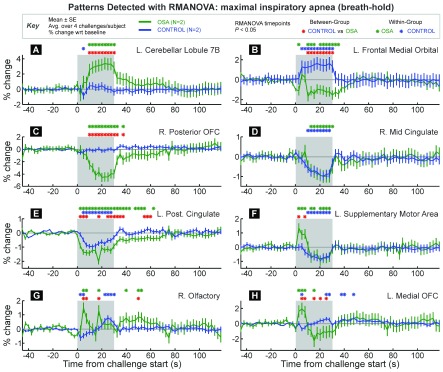
Average time trends for a control and patient group, averaged over four breath-hold challenges (30 s maximal inspiratory apnea), from eight VOI. VOI are from the Automatic Anatomical Labelling toolbox extension to SPM. Time-points of between and within-group significant responses relative to baseline period are indicated by color-coded “*” symbols. The graphs are in percent signal change relative to baseline, and each trace is a group/challenge average (± between-subject SE).

A set of results is presented in Figure 5, illustrating a variety of statistical responses for eight VOI within the same fMRI dataset. This section describes the analytic steps needed to arrive at these results.

***Residual tests of the RMANOVA assumptions.*** Several diagnostic tests were performed. The predicted responses were similar to the observed responses. Distributional tests of the Studentized residuals tests determined that the data were not precisely normally-distributed, as determined by the statistical tests with the PROC UNIVARIATE procedure. However, over 95% of the residuals were within ± 1.96, and visual inspection of the plots of residuals and predicted versus observed values showed only minor skew, and therefore the data were considered to be adequately modeled by the RMANOVA.

***Within and between-group responses highlighted by RMANOVA.*** We analyzed the fMRI signal responses. To infer neuronal responses that lead to the BOLD phenomenon that is measured by the fMRI signal, the patterns should be deconvolved with an HRF; this approach was not applied here.

The Tukey-Fisher criterion for multiple comparisons All VOI showed significance effects of the RMANOVA models at the global level ( Figure 5A–H), so in line with the Tukey-Fisher criterion for multiple comparisons, the independent variables and interactions were assessed. If any model was not significant at the global level, no further post-hoc assessments would have been performed, even if specific time-points showed significance. For the right mid cingulate ( Figure 5F), the group×time interaction was not significant, so no post hoc tests were performed on between-group time point effects. All VOI showed significant effects of “epoch” so within-group time points were assessed post hoc for all groups.

The three VOI in Figure 5A–C would likely all be highlighted as having significant group differences in response using conventional SPM analyses. However, RMANOVA provides more detail on when and how the groups differ. Figure 5A illustrates a typical fMRI pattern of difference, with one group showing increased signal throughout the challenge, and the other group essentially no change. Figure 5B illustrates an opposite response between the groups, with on increasing and the other decreasing during the challenge period. Figure 5C shows a decrease in one group with no change in the other.

Figure 5D illustrates a simple case of a similar change in both groups; thus the within-group timepoints of difference are highlighted during the challenge period, corresponding to an equivalent decrease in OSA and control subjects. Figure 5E shows both groups changing in the same direction, but with a greater magnitude of response in the OSA group, thus leading to significant between-group differences across multiple time-points. Figure 5E also illustrates a pattern of changes that lasts over 30 seconds into the recovery period.

Transient differences are shown in Figure 5F–H. Figure 5F shows a pattern of initial increased response in the OSA group and sustained decreased response in the control group. However, unlike previous examples, the trends in the OSA group change 10–15 seconds into the challenge from an increase to a decrease below baseline. Figure 5G shows a an opposite pattern in control subjects (initial decrease, latter increase), and in OSA two large peaks are identified as differing significantly both between group and within group relative to baseline. Finally, Figure 5H shows a switch between greater OSA signal at the start of the challenge followed by a lower signal during the second half of the task period. These examples illustrate the capability of RMANOVA to detect variable timing of within and between group differences in physiologic data.

## Discussion and conclusions

### Advantages of RMANOVA

The results illustrate two advantages of RMANOVA: firstly, no prior model of expected response of either signal intensity or timing is assumed, and for the type of challenge shown here, a variety of significant response patterns was detected by RMANOVA. Secondly, once a significant effect is found, RMANOVA provides an objective, statistically rigorous assessment of the time when responses or differences occurred, and the precise response pattern. A group difference highlighted by a traditional SPM analysis does not differentiate between a group increase vs. no change, a group increase vs. a decrease, no change vs. a decrease, or a larger increase vs. a smaller increase.

The procedure allows analysis of multiple subjects within multiple groups. The within-group and between-group are all performed with one analysis, allowing the contributions of subject and group factors to be accounted for in the final results.

Note that if all expected responses to the fMRI task are “on/off” or boxcar in nature, then the advantages of RMANOVA compared with cluster analysis are diminished.

Another advantage lies in processing timetrends from VOI drawn in different locations across different subjects. Spatial normalization is only accurate to within several millimeters ^18^, and therefore small brain structures, such as those in the brainstem cannot be accurately depicted on VOI across multiple spatially normalized scans. By allowing a group analysis of timetrends from varying sites, RMANOVA can be more accurate compared with conventional fMRI analyses in depicting group responses within a particular structure. For regions such as the dorsal medulla, VOI analysis using RMANOVA is particularly helpful for determining group patterns.

### Disadvantages of RMANOVA

Two disadvantages of RMANOVA relate to defining the VOI. The procedure requires manual definition of the VOI, perhaps for each subject, or at least for each structure of interest. A second disadvantage is that no whole-brain search is performed, and only *a priori* defined areas are studied. For this reason, we believe that a combination of a traditional SPM analysis with RMANOVA allows for a complete and powerful approach to analysis of fMRI data where the time-course of responses is of interest.

The approach does not consider or allow for variations in the shape of the HRF. A standard HRF may be used to deconvolve each time series, resulting in an inferred neural response time series, or a different HRF may be used for different VOI (to account for the spatial variation in HRF shape).

Temporal autocorrelation between repeated measures is not accounted for, which likely leads to some loss of power. In other words, the method does not account for the fact that responses at two adjacent time-points are more likely to be related than those at two separated time points. Nevertheless, we have found the technique highlights many patterns of interest, and thus this limitation does not negate the sensitivity of the technique. Theoretically, the limitation would be greater for signals that change slowly, such that adjacent time-points have very similar responses. In such a case, that is if smoother, less time-varying responses are expected, a model-based approach as in SPM or a time-series regression may be better suited to the analysis. Alternatively, for fMRI data in particular, pre-whitening could be applied to minimize the impact of temporal autocorrelations ^19^.

Procedural disadvantages with the proposed method include the requirement for detrending images prior to analysis, the extraction of the VOI time-trends from the images, and in the example presented here, the use of R or SAS. The former requires computation routines which are typically not included in fMRI analysis software, and the latter requires routines for exporting the data, availability of software, and some familiarity with this software.

There are a number of assumptions underlying the RMANOVA method, which are fully described above, along with their associated limitations. As with any statistical model, RMANOVA should be used with problems that match those assumptions.

## Conclusions

Analyzing timetrends from continuous physiologic measures using RMANOVA can highlight temporal patterns of response to a challenge not readily apparent using conventional model-based approaches. Our research group has used this method extensively to assess fMRI and cardio-respiratory responses to physiological challenges with complex responses over periods of tens of seconds to minutes. RMANOVA allows insight into the precise timing of changes from baseline, and response differences between groups. For complex paradigms, the technique can be a useful addition to conventional model-based approaches.

## Data and software availability

*F1000Research*: Dataset 1. Processed data, as well as the SAS code for running each VOI analysis, 10.5256/f1000research.8252.d117479 ^20^

*Harvard Dataverse*: Macey, Paul. 2016. Pilot fMRI of breath-hold in OSA, http://dx.doi.org/10.7910/DVN/EZUMI9 ^21^
