# Supplementary material for: Detecting variable responses in time-series using repeated measures ANOVA: Application to physiologic challenges
Source: F1000Res. 2016 Jul 8;5:563. Originally published 2016 Apr 5. [Version 2] doi: 10.12688/f1000research.8252.2 (PMC4995690; doi:10.12688/f1000research.8252.2)

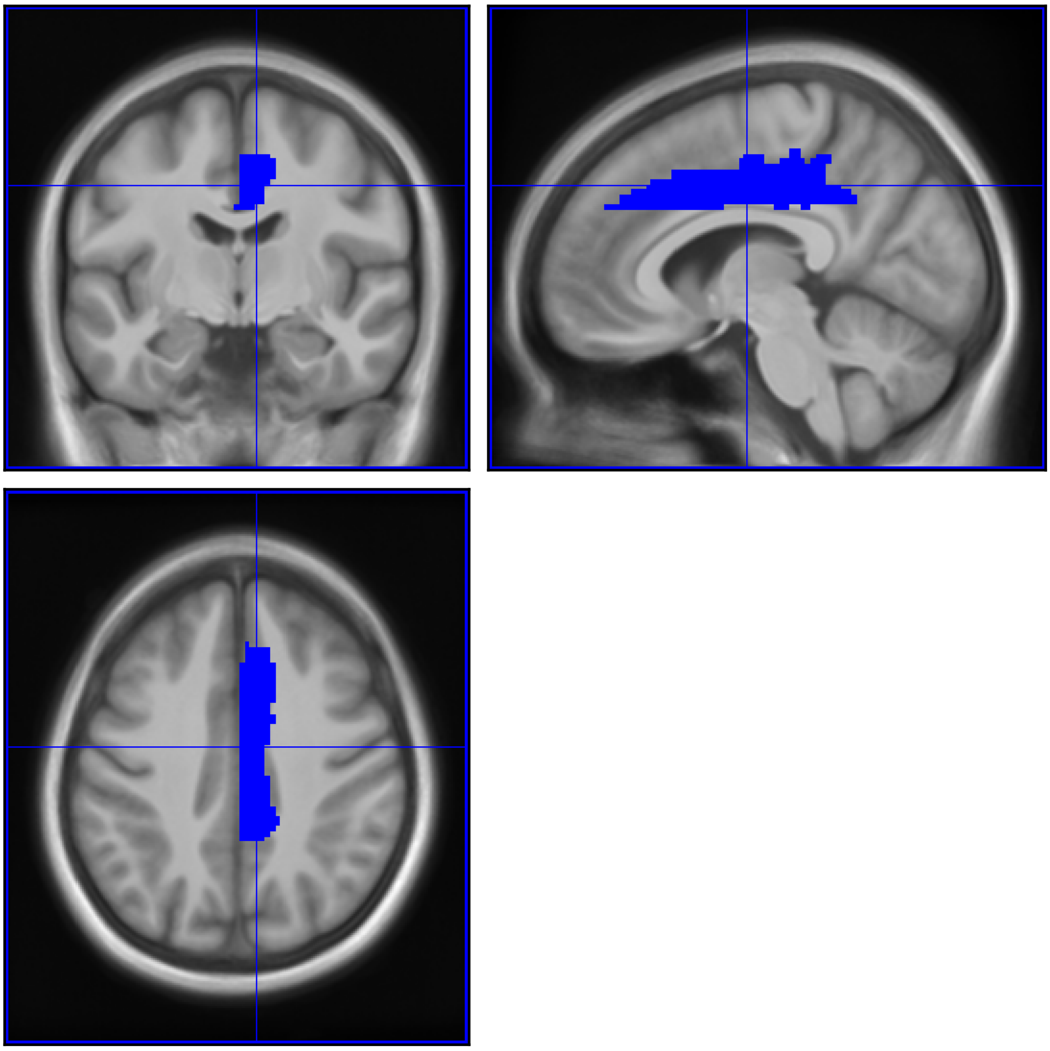

Supplement: Processed data, as well as the SAS code for running each VOI analysis — Organised as subfolders for each VOI, labeled by figure section. The VOI itself is also included as a nifti file, and in a pdf file overlaid onto a normalized background. [file f1000research-5-9880-s0000.tgz › ede87e86-7549-4b72-8795-051a7b317b6f_VOI.zip!/VOI/Fig 6D-CINMR/CINMR+.0f+8-10+.0f+38_disp.pdf]
