# Supplementary material for: Detecting variable responses in time-series using repeated measures ANOVA: Application to physiologic challenges
Source: F1000Res. 2016 Jul 8;5:563. Originally published 2016 Apr 5. [Version 2] doi: 10.12688/f1000research.8252.2 (PMC4995690; doi:10.12688/f1000research.8252.2)
Supplement: Peer review history

# Peer review: Referee response for version 2

**DOI:** 10.5256/f1000research.9880.r15277

**Published:** 2016-07-29

**Authors:** Matthew Brett

**Affiliations:** 1 Henry H. Wheeler, Jr. Brain Imaging Center, University of California, Berkeley, Berkeley, CA, USA

Thanks for the revisions. In particular, thanks for the R code, I think that will make the code from the paper much more accessible.

 I have some minor comments.

 In general, can you put the code in typewriter font or similar to differentiate the code and variable names from the rest of the text?

 Page 5 "Although other covariance matrices other than compound sym- metric might in theory be more suited to the present application, in our tests with SAS we found greatly increased computational time for minimal differences in results" - can you give any data on that conclusion?

 Top of page 6 "ROC SORT DATA= voilib.withingroup; BY group _epoch; RUN;The above code will create Withingroup and Betweengroup tables in the SAS library voilib."  I think the formatting got messed up?

 Page 7: "Violation of the homogeneity of covariance matrices generally results in the overall test having a higher Type I error rate than nominally set." - can you add a citation?

 Page 7-9: I might have missed it, but have you some guidance on the acceptable degree of not-normality or skewness?  For example, what do you mean by "moderate" or "serious" skew?

 Page 9 : Can you add citations for the statements on sphericity?

 Page 9: "we suggest a compound symmetric matrix" - can you give more justification for this choice?

 Page 9: "Smoothed images were intensity normalized to minimize global effects." - could you be specific what normalization you used?

 Page 10: "For the right mid cingulate (Figure 5F)," - I think you mean Figure 5D.

 Page 11: Have you previously defined the abbreviation OSA for Obstructive Sleep Apnea? 

 Page 11: "Temporal autocorrelation between repeated measures is not accounted for, which likely leads to some loss of power." - can you give more detail? Under what circumstances would temporal autocorrelation lead to excess false positives?

 Page 11: "Nevertheless, we have found the technique highlights many patterns of interest, and thus this limitation does not negate the sensitivity of the technique."  - maybe omit the second clause here? I guess it's possible that the effects you have here are so strong that they overwhelm an insensitive technique.

 Thanks again for the revisions.

I have read this submission. I believe that I have an appropriate level of expertise to confirm that it is of an acceptable scientific standard.

# Peer review: Referee response for version 2

**DOI:** 10.5256/f1000research.9880.r14836

**Published:** 2016-07-11

**Authors:** Joke Durnez

**Affiliations:** 1 Poldrack Lab, Department of Psychology, Stanford University, Stanford, CA, USA

I thank the authors for their careful revision. My comments and questions have been met and I deem this paper fit for indexation.

I have read this submission. I believe that I have an appropriate level of expertise to confirm that it is of an acceptable scientific standard.

# Peer review: Referee response for version 1

**DOI:** 10.5256/f1000research.8876.r13203

**Published:** 2016-05-04

**Authors:** Matthew Brett

**Affiliations:** 1 Henry H. Wheeler, Jr. Brain Imaging Center, University of California, Berkeley, Berkeley, CA, USA

This paper is shows how to apply the SAS "MIXED" procedure to FMRI data from ROIs, presents some data from this application, and discusses some of the options and diagnostics that this command provides.

The idea is to do what other packages would call an FIR analysis. You first decide on a time-bin width, typically one scan, but sometimes more. Call the time bin width B seconds. Then the scan data is labeled according to how many time bin widths it is after some event onset up until a cutoff of N time-bins.

Usually N * B is equal to the length of the event plus 20 seconds or so to let the hemodynamic response drop back to baseline. Then the fitting procedure calculates adjusted means for each for the N bins, giving an evoked response time-course that can simultaneously adjust for other effects in the data, fit in the regression model.

In what follows, I confess I know SPM much better than the other packages, so I'll mainly compare to SPM.

SPM is rather general, but it does have this capability. The standard procedure would be to fit an "FIR" model to each subject, giving an estimated signal for each time bin in beta images, with one beta image for each bin. You then take these time-bin beta images into your second-level model, where you can adjust for variance covariance effects such as correlation between time-bin values within subjects, different within-subject variance, and so on.

As you say, using these methods in SPM can be moderately hard, but I'd argue the complexity is comparable to switching languages from MATLAB to SAS and using SAS to calculate things like adjusted means, residuals and so on.

How would you expect the results of using the SPM interface (I guess within marsbar) to compare to the results of using the SAS methods you have here.

I was expecting to see you using either MATLAB or R to do this; MATLAB because SPM already requires MATLAB, and it's a general programming language, R because it has a variety of procedures for fitting repeated measures models (see for example http://www.r-statistics.com/2010/04/repeated-measures-anova-with-r-tutorials/), is free, open-source, and, in my experience, is more widely used than SAS in research

communities, including neuroimaging.

I'm afraid I haven't used SAS since the 1980s, so it's hard for me to comment on the details of the SAS commands.

I found myself skip-reading the actual SAS commands, I don't know how useful they will be for someone not experienced with SAS. Maybe in-line comments would help?

At various points you recommend options for diagnostics and outlier detection. It would help to have more detail as to why you recommend those options over others.

Description of procedure, first paragraph "Proportional scaling" is a technical term (at least in SPM-land) for dividing each scan voxel value by the calculated in-brain mean across voxels for the same scan. That is now very rarely used SPM, and was never widely used for FMRI.  The standard SPM scaling involves multiplying each scan in a run by the same scaling factor, that brings the mean of in-brain mean voxel values to 100.

**Assumptions, diagnostic checks, and limitations, first paragraph:**

Could you expand on "Violations of independence produce a non-normal distribution of the residuals"?

Options if diagnostic tests fail, first paragraph "but for smaller numbers the model is likely to be robust for no more than a small amount of skew.". Could you rephrase to something like "For small numbers, even a small amount of skew will make it unlikely that the model is robust.".  For some reason I found your original sentence a bit difficult to parse.

**Disadvantages of RMANOVA, third paragraph:**

You mention that "Temporal autocorrelation between repeated measures is not accounted for".  Could you elaborate? I guess you mean that repeated measures variance / covariance adjustments in MIXED are for the estimates across time bins, not for the autocorrelation between scans. In that case, are you

expecting something like SPM to do better, given that it does adjust for autocorrelation at the first level?

I have read this submission. I believe that I have an appropriate level of expertise to confirm that it is of an acceptable scientific standard, however I have significant reservations, as outlined above.

# Response

**Published:** 2016-07-01

**Authors:** Paul Macey

**Affiliations:** UCLA, USA

[Initial comments repeated for both reviewers]

 Thanks to the reviewers for their thoughtful comments. We made extensive revisions to the paper, which should make this method more accessible. The major changes including adding R code for implementing RMANOVA and diagnostic tests, simplifying some of the SAS commands and formatting requirements, and clarifying the nature of time series for which the method is applicable.

 We did investigate implementing the method in MATLAB, but found that approach to be cumbersome and limited. While we did not test the method in SPSS, our understanding is that such implementation is similar to SAS, and relatively straight forward. In R, RMANOVA is non-trivial, and we settled on a mixed model approach that is equivalent to the one presented in SAS, but with more limited options should the user wish to try alternative covariance options. Furthermore, in R there are many options for diagnostic plots and calculations, and we selected one series of commands and packages that implement all SAS outputs other than studentized residuals.

 In proposing a simplified data format, we decided to expand the scope of the method to include physiologic signals other than fMRI, such as heart rate, pulse oximetry, and other continuously acquired signals. We have applied our RMANOVA to these other signals in several publications. We therefore changed the title of the paper to reflect this broader application.

 We updated the “dataverse” repository to include example R and SAS code, and a formatted data file.

**Specific responses to Matthew Brett's comments**

 The reviewer’s comments are helpful in clarifying how this method fits with other available approaches.

- *This paper shows how to apply the SAS "MIXED" procedure to FMRI data from ROIs, presents some data from this application, and discusses some of the options and diagnostics that this command provides.*
*The idea is to do what other packages would call an FIR analysis. You first decide on a time-bin width, typically one scan, but sometimes more. Call the time bin width B seconds. Then the scan data is labeled according to how many time bin widths it is after some event onset up until a cutoff of N time-bins.*
Usually N * B is equal to the length of the event plus 20 seconds or so to let the hemodynamic response drop back to baseline. Then the fitting procedure calculates adjusted means for each for the N bins, giving an evoked response time-course that can simultaneously adjust for other effects in the data, fit in the regression model.
*In what follows, I confess I know SPM much better than the other packages, so I'll mainly compare to SPM.*
*SPM is rather general, but it does have this capability. The standard procedure would be to fit an "FIR" model to each subject, giving an estimated signal for each time bin in beta images, with one beta image for each bin. You then take these time-bin beta images into your second-level model, where you can adjust for variance covariance effects such as correlation between time-bin values within subjects, different within-subject variance, and so on.*
*As you say, using these methods in SPM can be moderately hard, but I'd argue the complexity is comparable to switching languages from MATLAB to SAS and using SAS to calculate things like adjusted means, residuals and so on.*
  An SPM FIR analysis could replicate the SAS implementation of our timetrend RMANOVA approach, and we have used FIR in single-group fMRI studies of physiologic challenges. However, the results of a multi-group FIR quickly become complex since there is a voxel-level map of significant responses at each time point during the challenge and recovery. Performing between-group comparisons with FIR requires quite large and complex design matrices and contrasts. The marsbar toolbox allows extraction of the timetrend for those contrasts at pre-determined VOI, but to indicate timepoints of significant within or between group responses, the user would still need to identify these manually from the bin contrast maps (at least that is my understanding).
  We agree that SAS is a complex environment unfamiliar to most neuroimaging researchers. At the time we originally developed this approach, MATLAB and R did not have the necessary capabilities, but that has mostly changed. In our revised paper, we present an implementation in R. We evaluated MATLAB but found no reasonable way to implement the present type of RMANOVA. In practice, the method is automated, so users don’t need to work within R or SAS other than running the analyses.
  One advantage of SAS over R is the option to easily choose a variety of covariance matrices. This could be important for experiments where the variability changes over time. For example, the standard “compound symmetric” (cs) covariance matrix we propose assumes equal variability at all timepoints including before during and after a task, whereas, for example, the “compound symmetric heterogenous” (csh) covariance matrix allows for different variances (and hence standard error estimates) at each timepoint. Theoretically csh should allow for more accurate models, although we found no major differences for our physiologic experiments and in our example use cs due to the lower computation needs. The R implementation does not have the option to choose covariance matrices, but we used a random intercept for each subject, which is equivalent to cs.
- *How would you expect the results of using the SPM interface (I guess within marsbar) to compare to the results of using the SAS methods you have here.*
  The principal advantage of our technique is that it gives simple results that match the fMRI time-trend patterns. If a timetrend is higher or lower than the baseline at a timepoint, the method will indicate whether that increase was significant or not via a timepoint-specific p-value. If the timetrends for two groups diverge at a timepoint, the method will indicate whether that group difference is significant, again with a timepoint-specific p value.
- *I was expecting to see you using either MATLAB or R to do this; MATLAB because SPM already requires MATLAB, and it's a general programming language, R because it has a variety of procedures for fitting repeated measures models (see for example* *http://www.r-statistics.com/2010/04/repeated-measures-anova-with-r-tutorials/* *), is free, open-source, and, in my experience, is more widely used than SAS in research* *communities, including neuroimaging.*
*I'm afraid I haven't used SAS since the 1980s, so it's hard for me to comment on the details of the SAS commands.*
*I found myself skip-reading the actual SAS commands, I don't know how useful they will be for someone not experienced with SAS. Maybe in-line comments would help?*
  We have provided an R implementation, and added comments.
- *At various points you recommend options for diagnostics and outlier detection. It would help to have more detail as to why you recommend those options over others.*
  We have added additional comments explaining the tests.
- *Description of procedure, first paragraph "Proportional scaling" is a technical term (at least in SPM-land) for dividing each scan voxel value by the calculated in-brain mean across voxels for the same scan. That is now very rarely used SPM, and was never widely used for FMRI.  The standard SPM scaling involves multiplying each scan in a run by the same scaling factor that brings the mean of in-brain mean voxel values to 100.*
  We have removed this comment.

**Assumptions, diagnostic checks, and limitations, first paragraph:**

- *Could you expand on "Violations of independence produce a non-normal distribution of the residuals"?*
  We added some further explanation of independence violations.
- *Options if diagnostic tests fail, first paragraph "but for smaller numbers the model is likely to be robust for no more than a small amount of skew.". Could you rephrase to something like "For small numbers, even a small amount of skew will make it unlikely that the model is robust.". For some reason I found your original sentence a bit difficult to parse.*
  We agree the original wording was confusing, and we have reworded according to this suggestion.

**Disadvantages of RMANOVA, third paragraph:**

- *You mention that "Temporal autocorrelation between repeated measures is not accounted for".  Could you elaborate? I guess you mean that repeated measures variance / covariance adjustments in MIXED are for the estimates across time bins, not for the autocorrelation between scans. In that case, are you expecting something like SPM to do better, given that it does adjust for autocorrelation at the first level?*
  We give more detail in this paragraph. Specifically, we note that if there is a high correlation between adjacent time points (that is, there is only slow variation), this limitation will be more prominent. If the response are not expected to change much over time, SPM would be a better approach. We try to capture this aspect with “variable responses” in the paper title.

 

# Peer review: Referee response for version 1

**DOI:** 10.5256/f1000research.8876.r13288

**Published:** 2016-04-26

**Authors:** Joke Durnez

**Affiliations:** 1 Poldrack Lab, Department of Psychology, Stanford University, Stanford, CA, USA

This paper aims to present a different approach to analyzing fMRI time trends.  The idea is to average the signal overall trials, over subjects (similar to ERP-analysis) and look at the differences between groups and conditions and especially its interaction with time since onset. Because the trials are clustered within subjects, the data points are modeled using a mixed model.  

The result is a figure with the average time course per group/condition, with a statistical test comparing the BOLD signal at each time point.

The main difference between this model and the model in the standard (frequentist, parametric mixed model) procedure is the specific within-subjects design matrix. The predictor over time in a standard analysis would be:

[ 0 0 0 1 1 1 0 0 0 1 1 1] o HRF

The predictor over time with the presented approach would be a categorical variable:

[ 0 0 0 1 2 3 0 0 0 1 2 3 ]

The model tested is then BOLD = GROUP + TIME + GROUP*TIME.  Important: TIME is modeled as a categorical variable, not continuous. Modeling time as a categorical folder allows to test for each time point separately as a post-hoc test. The results of interest are the post-hoc tests of the interaction effect between group and time.

SOME COMMENTS

1. The focus of the paper is on the post-hoc tests.  These are definitely interesting, but the post-hoc tests should only be performed if the interaction group*time is significant. It would be interesting to also have some more detail on the main model (e.g. main effect of time, what does it mean? what is the interaction?)
2. In my opinion the chunks of code make it hard to read through the paper. It would be easier if the methods section only describes the different choices/tests/parameters and the code is grouped in an appendix. Especially code that is just for the purpose of making variables or figures should not be in the main manuscript.
3. In general, I think SAS is a bad choice. Even though it has definitely one of the best mixed model implementations, it’s not a typical language at all in neuroimaging. If you want people to use this procedure, consider having a script in R, matlab or python.
4. I have tried to reproduce the code with the data attached. However, the datasets have different names and paths. It’s hard to derive what is what…
5. I very much liked the diagnostics, something that is most often lacking in neuroimaging studies.
6. I think the part about missing data could be removed. This problem is non-existing in fMRI.
7. In the results section: “all significant effects were BOLD signal responses”. Poor choice of words. The outcome variable is the BOLD signal response, not the effect.
8. “the highlighted VOI did show significant overall effects”: significant overall effects of group? of time? the interaction? all of them?
9. The interpretation given to the time series is the interpretation of a pattern.  However, these tests are not tests for patterns. For example, the interpretation of the interaction on fig 6E is not significant on all time points but is interpreted as such. This door is wide open for over interpreting non-significant results. This is a problem with these point wise comparisons.
10. There are ways to take away the temporal autocorrelation (whitening), which is currently used in fMRI software. You should mention this when talking about the problem of temporal correlation.

I have read this submission. I believe that I have an appropriate level of expertise to confirm that it is of an acceptable scientific standard, however I have significant reservations, as outlined above.

# Response

**Published:** 2016-07-01

**Authors:** Paul Macey

**Affiliations:** UCLA, USA

[Initial comments both reviewers]

 Thanks to the reviewers for their thoughtful comments. We made extensive revisions to the paper, which should make this method more accessible. The major changes including adding R code for implementing RMANOVA and diagnostic tests, simplifying some of the SAS commands and formatting requirements, and clarifying the nature of time series for which the method is applicable.

 We did investigate implementing the method in MATLAB, but found that approach to be cumbersome and limited. While we did not test the method in SPSS, our understanding is that such implementation is similar to SAS, and relatively straight forward. In R, RMANOVA is non-trivial, and we settled on a mixed model approach that is equivalent to the one presented in SAS, but with more limited options should the user wish to try alternative covariance options. Furthermore, in R there are many options for diagnostic plots and calculations, and we selected one series of commands and packages that implement all SAS outputs other than studentized residuals.

 In proposing a simplified data format, we decided to expand the scope of the method to include physiologic signals other than fMRI, such as heart rate, pulse oximetry, and other continuously acquired signals. We have applied our RMANOVA to these other signals in several publications. We therefore changed the title of the paper to reflect this broader application.

 We updated the “dataverse” repository to include example R and SAS code, and a formatted data file.

**Specific responses to Joke Durnez's comments**

*This paper aims to present a different approach to analyzing fMRI time trends.  The idea is to average the signal overall trials, over subjects (similar to ERP-analysis) and look at the differences between groups and conditions and especially its interaction with time since onset. Because the trials are clustered within subjects, the data points are modeled using a mixed model.*

*The result is a figure with the average time course per group/condition, with a statistical test comparing the BOLD signal at each time point.*

**

*The main difference between this model and the model in the standard (frequentist, parametric mixed model) procedure is the specific within-subjects design matrix. The predictor over time in a standard analysis would be:*

*[ 0 0 0 1 1 1 0 0 0 1 1 1] o HRF*

**

*The predictor over time with the presented approach would be a categorical variable:*

*[ 0 0 0 1 2 3 0 0 0 1 2 3 ]*

The model tested is then BOLD = GROUP + TIME + GROUP*TIME.  Important: TIME is modeled as a categorical variable, not continuous. Modeling time as a categorical folder allows to test for each time point separately as a post-hoc test. The results of interest are the post-hoc tests of the interaction effect between group and time.

 We appreciate this simplified description of the technique; we have included a synthesis at the start of the Introduction.

 SOME COMMENTS

1. The focus of the paper is on the post-hoc tests.  These are definitely interesting, but the post-hoc tests should only be performed if the interaction group*time is significant. It would be interesting to also have some more detail on the main model (e.g. main effect of time, what does it mean? what is the interaction?)
  The sequence is to test 1) global fit of the model; if significant then test 2) variable and interaction level fit; and if significant test 3) post hoc at individual time points. This sequence was not clear in the original version. We agree that pot hoc assessments at time points are only valid for significant model-level effects of time (within group) or group*time (between group), and we have emphasized this point in the revised paper. 
2. *In my opinion the chunks of code make it hard to read through the paper. It would be easier if the methods section only describes the different choices/tests/parameters and the code is grouped in an appendix. Especially code that is just for the purpose of making variables or figures should not be in the main manuscript. *
  We have simplified the code and proposed input data format, which eliminates some of the more cumbersome and less relevant code. We did originally have the code in appendices, but this format did not fit the requirements of this journal. In the revised paper, we added comments and restructured some sections to improve readability.
3. *In general, I think SAS is a bad choice. Even though it has definitely one of the best mixed model implementations, it’s not a typical language at all in neuroimaging. If you want people to use this procedure, consider having a script in R, matlab or python.*
  We now provide an implementation in R, since this software is free and well supported in the statistical community. We did evaluate RMANOVA in MATLAB, but found no reasonable approach to replication the implementation we propose.
  While SAS is an environment principally familiar to statisticians, the implementation of the procedure and diagnostic tests is simpler and more comprehensive than R. For that reason, we have left the SAS code in the paper.
4. *I have tried to reproduce the code with the data attached. However, the datasets have different names and paths. It’s hard to derive what is what…*
  We simplified the input data format to be a text file with three or four columns. The original approach of importing from Excel was cumbersome, but with the more logical format users should find it easier to run the SAS or R commands.  
5. *I very much liked the diagnostics, something that is most often lacking in neuroimaging studies.*
  We appreciate the comment. We have added explanations, as noted in response to Matthew Brett.
6. *I think the part about missing data could be removed. This problem is non-existing in fMRI.*
  As noted in response to Matthew Brett , we have expanded the paper to explain how this RMANOVA approach applies to non-fMRI physiologic data, where missing values are possible. We have clarified the limitation.
7. *In the results section: “all significant effects were BOLD signal responses”. Poor choice of words. The outcome variable is the BOLD signal response, not the effect.*
  We revised this introductory paragraph. 
8. *“the highlighted VOI did show significant overall effects”: significant overall effects of group? of time? the interaction? all of them?*
  We revised the wording in this section.
9. *The interpretation given to the time series is the interpretation of a pattern.  However, these tests are not tests for patterns. For example, the interpretation of the interaction on fig 6E is not significant on all time points but is interpreted as such. This door is wide open for over interpreting non-significant results. This is a problem with these point wise comparisons.*
  The reviewer is correct to note RMANOVA is not identifying patterns, but rather effects at time points independent of each other. We have changed this terminology.
  We have emphasized that the time points will only be assessed post hoc if the corresponding independent variable is significant within the model. If the group×time interaction is not significant, the between-group time points will not be considered. If the time variable is not significant, the within-group time points will not  be considered.
10. *There are ways to take away the temporal autocorrelation (whitening), which is currently used in fMRI software. You should mention this when talking about the problem of temporal correlation.*
  We have expanded this paragraph and included a note about whitening.
